# A Combined Therapy with Myo-Inositol and D-Chiro-Inositol Improves Endocrine Parameters and Insulin Resistance in PCOS Young Overweight Women

**DOI:** 10.1155/2016/3204083

**Published:** 2016-07-14

**Authors:** Elena Benelli, Scilla Del Ghianda, Caterina Di Cosmo, Massimo Tonacchera

**Affiliations:** Department of Clinical and Experimental Medicine, Section of Endocrinology, University Hospital of Pisa, Via Paradisa 2, 56124 Pisa, Italy

## Abstract

*Introduction.* We evaluated the effects of a therapy that combines myo-inositol (MI) and D-chiro-inositol (DCI) in young overweight women affected by polycystic ovary syndrome (PCOS), characterized by oligo- or anovulation and hyperandrogenism, correlated to insulin resistance.* Methods.* We enrolled 46 patients affected by PCOS and, randomly, we assigned them to two groups, A and B, treated, respectively, with the association of MI plus DCI, in a 40 : 1 ratio, or with placebo (folic acid) for six months. Thus, we analyzed pretreatment and posttreatment FSH, LH, 17-beta-Estradiol, Sex Hormone Binding Globulin, androstenedione, free testosterone, dehydroepiandrosterone sulphate, HOMA index, and fasting glucose and insulin.* Results.* We recorded a statistically significant reduction of LH, free testosterone, fasting insulin, and HOMA index only in the group treated with the combined therapy of MI plus DCI; in the same patients, we observed a statistically significant increase of 17-beta-Estradiol levels.* Conclusions.* The combined therapy of MI plus DCI is effective in improving endocrine and metabolic parameters in young obese PCOS affected women.

## 1. Introduction

Polycystic ovary syndrome (PCOS) is a heterogeneous syndrome, involving a growing number of women in reproductive age, diagnosed on the basis of three different factors: oligo- or anovulation, clinical/biochemical hyperandrogenism, and polycystic ovary, with the presence on ultrasound of ≥12 follicles in each ovary measuring 2 ± 9 mm in diameter and/or increased ovarian volume (>10 mL) [1, 2]. PCOS affected patients that had menstrual irregularity, followed in many cases by infertility [3, 4] and mood disorders, such as anxiety and depression [5]. Though PCOS pathogenesis still remains unclear, insulin resistance (IR) and the consequential hyperinsulinemia are considered primary triggers, both in obese and in lean women with this syndrome [6–8]. Indeed, hyperinsulinemia induced by IR occurs in roughly 80% of PCOS obese women, as in 30–40% of PCOS lean women [9], suggesting that IR is independent but also exacerbated by obesity; this latter considered an enhancing factor that positively correlates with the multifactorial syndrome [10–12]. It was hypothesized that in patients with PCOS altered insulin signaling may generate the IR which in turn causes abnormal ovarian steroidogenesis [13, 14], so that several insulin-sensitizing compounds have been proposed as possibly safe and efficacious long-term treatment of PCOS [15]. Among these drugs, metformin resulted to be the most used and studied drug, even if this molecule is predominantly associated with gastrointestinal discomforts consisting of bloating, nausea, and diarrhea [14, 16]. Interesting and promising results have been obtained focusing on two inositol stereoisomers, such as myo-inositol (MI) and D-chiro-inositol (DCI), acting like insulin mediators [17–19]. As insulin second messengers, both these molecules are involved in increasing insulin sensitivity of different tissues to improve metabolic and ovulatory functions [20]. In particular, at low dosage, DCI restores normal insulin sensitivity in the typical insulin target tissues, reducing the circulating insulin and androgens and inducing an enhancement in ovulation frequency. On the contrary, MI exerts its beneficial effects mainly at the ovary level, where it is highly concentrated, both enhancing insulin pattern and also directly acting on a number of ovarian functions, including steroidogenesis [21]. Some authors [22] postulated that PCOS affected women, with IR, presenting in the ovary a misbalance in MI/DCI ratio, resulting in DCI overproduction and in turn in a deficiency in MI, which would explain the excessive androgen biosynthesis. Other authors [23], instead, proposed that the increased androgen levels in PCOS patients might be linked to a decreased MI/DCI. In a recent study, Facchinetti et al. [24] discovered that the physiological MI/DCI ratio was 40 : 1 and, based on this finding, as well as on the specific behavior of both stereoisomers, we investigated the effects of a therapy that combines MI plus DCI in the ratio of 40 : 1, versus placebo, in order to improve some clinical outcomes in PCOS young overweight women.

## 2. Methods

### 2.1. Patients and Study Design

This randomized controlled trial enrolled 46 obese women with BMI > 30 who were affected by PCOS according to Rotterdam criteria [1, 2]. All the women were enrolled at the Department of Clinical and Experimental Medicine, University of Pisa. Patients with diabetes, smokers, and alcohol users were ruled out from the study. After all patients subscribed their written informed consent to be involved into the study, they were randomly assigned to two groups, A and B. At baseline, patients in groups A and B did not differ significantly. In group A, 21 women received MI plus DCI combined treatment at the ratio of 40 : 1 (the physiologic ratio of the two isomers in the body) in soft gel capsule containing 550 mg of MI, 13.8 mg of DCI, and 200 *μ*g of folic acid (INOFOLIC® COMBI, LO.LI.PHARMA) twice a day. Group B, with 25 women, received the same amount of folic acid (200 *μ*g) as placebo twice a day. The treatments were performed for six months. At the beginning of the study, all the patients were in the follicular phase of the menstrual cycle.

### 2.2. Study Measurements

All patients were evaluated for FSH, LH, 17-beta-Estradiol (E), Sex Hormone Binding Globulin (SHBG), androstenedione, free testosterone, and dehydroepiandrosterone sulphate (DHEAS) levels at the baseline and after the six months of therapy with MI plus DCI association or with placebo. FSH and LH serum levels were detected by immune-enzymatic assay (Access Immunoassay System, hLH, hFSH, Beckman Coulter, Brea, CA, USA). Estradiol levels were measured by competitive immunoassay (Access Immunoassay System, Estradiol, Beckman Coulter, Brea, CA, USA). SHBG levels were detected by immunoassay (Access Immunoassay System, SHBG, Beckman Coulter, Brea, CA, USA). Serum levels of androstenedione were measured by conventional immune-enzymatic assay (Access Immunoassay System, androstenedione, Beckman Coulter, Brea, CA, USA). Free testosterone serum levels were measured by immune-enzymatic assay (Access Immunoassay System, free testosterone, Beckman Coulter, Brea, CA, USA). DHEAS was measured by conventional immunoassay (Access Immunoassay System, DHEAS, Beckman Coulter, Brea, CA, USA). Insulin resistance was measured by means of Homeostasis Model Assessment (HOMA) in addition to determining fasting glucose and insulin with the same timeline and modalities. Blood samples, taken at the baseline and after the six-month treatment period under similar conditions, were separated by centrifugation at 2000 ×g for 15 minutes at 4°C, and the serum obtained was stored at −20°C within one hour of collection. Before the analysis, all the serum samples were thawed and entirely mixed.

### 2.3. Statistical Analysis

Data reported indicate mean values ± standard deviation (SD). Paired *t*-test was used to identify the differences between variables at baseline and after six months of treatment with MI plus DCI or with placebo, respectively. Differences were considered statistically significant at *p* value <0.05.

## 3. Results and Discussion

The goal of this study was to investigate if the therapy combining MI and DCI in the ratio of 40 : 1 could improve the endocrine profile and the insulin resistance of obese women with a PCOS diagnosis. To address this issue, 46 young obese patients affected by this syndrome, whose characteristics are summarized in Table 1, were randomly included in two groups and then treated with MI plus DCI at the ratio of 40 : 1 with or placebo for six months. Insulin resistance, evaluated as HOMA index, fasting insulin, and fasting glucose, and also hormonal parameters were determined at the baseline and after the six-month therapy. As shown in Table 2, we observed that, with respect to the baseline values, only the combined therapy of MI plus DCI significantly rebalanced the endocrine and metabolic profiles of these patients, ameliorating their insulin resistance and the ovulatory function, as successfully recorded by ultrasound. As a matter of fact, LH and free testosterone levels decreased after the combined treatment, downregulating the hyperandrogenism, and even HOMA index and fasting insulin, markers of insulin resistance, resulted to be significantly reduced. On the other hand, E and SHBG significantly increased, showing restoring in ovulation capability. No relevant changes in these sex hormones were reported in group B, treated with placebo, and no significant modifications were observed after the treatment in both groups A and B for what concerns BMI, FSH, androstenedione, DHEAS, and fasting glucose. Importantly, no relevant side effect was recorded during the combined therapy with MI plus DCI. Overall, these results demonstrated the clinical importance of a combined therapy of MI plus DCI to correct the PCOS metabolic and reproductive aspects and they are largely in agreement with the issues discussed on the two international consensus conferences on MI, DCI, and their link with PCOS [25, 26]. PCOS is a syndrome whose pathogenesis remains still largely unclear, even though several etiological factors are demonstrated to be involved. Compelling evidences claimed the pivotal role of insulin resistance and/or compensatory hyperinsulinemia in this syndrome [9, 27–29]; indeed they tightly contribute both directly (increasing the ovarian production of androgens) and indirectly (modulating the hepatic SHBG synthesis) to hyperandrogenism development, one of the main features of those patients affected by PCOS [30, 31], especially in case of overweight women [32]. Nevertheless, literature findings consistently demonstrated that a deficiency in the tissue availability and/or usage of MI and/or DCI in women diagnosed with PCOS could likely concur to the IR typical of this syndrome [22, 23]. The two inositol stereoisomers, MI and DCI, acting as insulin-sensitizers, have been demonstrated to positively influence the clinical history of PCOS patients, ameliorating their endocrine and metabolic profile both alone and in combination [19, 33–37]. DCI alone, at low dosage, may restore normal insulin sensitivity in the typical insulin target tissues, inducing an enhancement in ovulation frequency which could be ascribed to the general improved insulin sensitivity and to the reduced circulating insulin and androgens. On the contrary, MI exerts its beneficial effects mainly at the ovary level, both enhancing insulin pattern and also directly acting on a number of ovarian functions, including steroidogenesis [22]. The ability of both inositol stereoisomers to regulate glucose metabolism in a different manner (DCI promotes glycogen synthesis, while MI may support glucose cell intake) [38] is mirrored by their different concentration in the tissues: while DCI is highly concentrated in glycogen storage tissues (liver, muscles, and fat), MI is more abundant in those tissues that need a large amount of glucose, such as brain, heart, or ovary [39]. From this knowledge, a combined therapy with MI plus DCI in their physiological plasma ratio (MI/DCI 40 : 1) seems to be the most appropriated clinical approach to integrate the positive effects exerted by both inositol stereoisomers.

## 4. Conclusions

The data reported are encouraging and they offer therapeutic options to the first-line treatments in PCOS women with moderate or severe hyperandrogenism and/or menstrual abnormalities, which are represented by metformin as well as by oral contraceptives. These compounds effectively suppress LH release and the consequent androgen production from the ovary; also they increase the sex hormone binding protein synthesis, lowering the levels of circulating free androgens [40]. Unfortunately, if the patient aims to restore ovulation in order to conceive, contraceptives are not the clinical strategy to follow. Furthermore, prolonged use of contraceptives may increase homocysteine levels after six months of treatment [41], as well as the risk of venous thromboembolism [42]. For what concerns metformin, several side gastrointestinal effects (diarrhoea, nausea, vomiting, and abdominal bloating) and metabolic complications have been evidenced after a long-term treatment [43]. For all these reasons, even though more studies on a higher number of patients and with greater statistical significance are needed to confirm these striking posttreatment outcomes, safe combined use of inositol stereoisomers should be largely suitable and it might represent a valid clinical approach in PCOS management.

## Figures and Tables

**Table 1:** Characteristics of patients who received MI plus DCI (group A) or placebo treatment (group B).

	Group A (*n* = 21)	Group B (*n* = 25)
Age (years)	23 ± 6.8	25 ± 7.3
Height (cm)	164 ± 6.7	168 ± 6.9
Weight (kg)	85 ± 13.5	88 ± 14
BMI	32 ± 4.8	31 ± 4.6

BMI: body mass index.

**Table 2:** Baseline and posttreatment endocrine and metabolic parameters of groups A and B of PCOS patients.

	Group A (*n* = 21)			Group B (*n* = 25)		
	Baseline	MI plus DCI	*p* value	Baseline	placebo	*p* value
FSH (mIU/mL)	5.86 ± 1.75	4.96 ± 1.74	ns	5.67 ± 1.11	5.47 ± 0.63	ns
LH (mIU/mL)	12.5 ± 8	8.5 ± 4.04	*p* < 0.05	11.27 ± 7.2	11.25 ± 5.35	ns
E (pg/mL)	47.06 ± 18.20	107.42 ± 92.86	*p* < 0.01	50.37 ± 19.45	52 ± 20.2	ns
Fasting insulin (*μ*U/mL)	20.19 ± 8.14	10.74 ± 5.46	*p* < 0.001	18 ± 8	17.8 ± 8.2	ns
Fasting glucose (mg/dL)	85 ± 5.96	86 ± 7.12	ns	86.2 ± 9.1	84.73 ± 8.3	ns
Free testosterone (ng/dL)	0.76 ± 0.20	0.62 ± 0.15	*p* < 0.05	0.85 ± 0.22	0.83 ± 0.2	ns
SHBG (nmol/L)	24.11 ± 10.35	35.85 ± 24.3	*p* < 0.05	20.44 ± 8.77	21.36 ± 7.57	ns
Androstenedione (ng/mL)	4.25 ± 1.48	4.01 ± 1.70	ns	3.48 ± 1.21	3.12 ± 2.23	ns
DHEAS (*μ*g/dL)	327.32 ± 150.89	347.6 ± 170.98	ns	337.95 ± 155.79	315.83 ± 145.59	ns
HOMA	3.38 ± 1.97	1.97 ± 1.48	*p* < 0.05	3.48 ± 2.02	2.8 ± 1.4	ns

E, 17-beta-Estradiol; P, progesterone; 17OHP, 17-OH-progesterone; SHBG, Sex Hormone Binding Globulin; DHEAS, dehydroepiandrosterone sulphate.

## Abbreviations

DCI: D-Chiro-inositol
DHEAS: Dehydroepiandrosterone sulphate
E: 17-Beta-Estradiol
HOMA: Homeostasis Model Assessment
IR: Insulin resistance
MI: Myo-inositol
PCOS: Polycystic ovary syndrome
SHBG: Sex Hormone Binding Globulin.
